# The Enterics for Global Health (EFGH) *Shigella* Surveillance Study in Malawi

**DOI:** 10.1093/ofid/ofae050

**Published:** 2024-03-25

**Authors:** Donnie Mategula, Maureen Ndalama, Clement Lefu, Jobiba Chinkhumba, Latif Ndeketa, Vitumbiko Munthali, Clifford Chitala, Thandizo Malemia, Gertrude Million, Ishmail Mbutuka, Ranken Mhone, Ethel Makwenda, Mussa James, Cornelius Bwanali, Gift Kazembe, Abell Manundo, Evance Chauluka, Salama Chitalo, Ethel Alumando, Dalitso Longwe, Maggie Matandika, Paul Jonasi, Agra Thindwa, Deborah Phiri, Richard Wachepa, Flywell Kawonga, Victor Maiden, Mary Charles, Ida Kapindula, Desiree Witte, Ann M Turner, Christina Bronowski, Kate Baker, Naor Bar-Zeev, Melita A Gordon, Queen Dube, Nigel A Cunliffe, Khuzwayo C Jere, Jennifer Cornick

**Affiliations:** Malawi Liverpool Wellcome Programme, Blantyre, Malawi; Clinical Sciences Department, Liverpool School of Tropical Medicine, Liverpool, United Kingdom; Department of Environmental and Community Health, School of Global Public Health, Kamuzu University of Health Sciences, Blantyre, Malawi; Malawi Liverpool Wellcome Programme, Blantyre, Malawi; Malawi Liverpool Wellcome Programme, Blantyre, Malawi; Department of Environmental and Community Health, School of Global Public Health, Kamuzu University of Health Sciences, Blantyre, Malawi; Malawi Liverpool Wellcome Programme, Blantyre, Malawi; Department of Environmental and Community Health, School of Global Public Health, Kamuzu University of Health Sciences, Blantyre, Malawi; Institute of Infection, Veterinary and Ecological Sciences, University of Liverpool, Liverpool, United Kingdom; Malawi Liverpool Wellcome Programme, Blantyre, Malawi; Malawi Liverpool Wellcome Programme, Blantyre, Malawi; Malawi Liverpool Wellcome Programme, Blantyre, Malawi; Malawi Liverpool Wellcome Programme, Blantyre, Malawi; Malawi Liverpool Wellcome Programme, Blantyre, Malawi; Malawi Liverpool Wellcome Programme, Blantyre, Malawi; Malawi Liverpool Wellcome Programme, Blantyre, Malawi; Malawi Liverpool Wellcome Programme, Blantyre, Malawi; Malawi Liverpool Wellcome Programme, Blantyre, Malawi; Malawi Liverpool Wellcome Programme, Blantyre, Malawi; Malawi Liverpool Wellcome Programme, Blantyre, Malawi; Malawi Liverpool Wellcome Programme, Blantyre, Malawi; Malawi Liverpool Wellcome Programme, Blantyre, Malawi; Malawi Liverpool Wellcome Programme, Blantyre, Malawi; Malawi Liverpool Wellcome Programme, Blantyre, Malawi; Malawi Liverpool Wellcome Programme, Blantyre, Malawi; Malawi Liverpool Wellcome Programme, Blantyre, Malawi; Malawi Liverpool Wellcome Programme, Blantyre, Malawi; Malawi Liverpool Wellcome Programme, Blantyre, Malawi; Malawi Liverpool Wellcome Programme, Blantyre, Malawi; Malawi Liverpool Wellcome Programme, Blantyre, Malawi; Malawi Liverpool Wellcome Programme, Blantyre, Malawi; Malawi Liverpool Wellcome Programme, Blantyre, Malawi; Malawi Liverpool Wellcome Programme, Blantyre, Malawi; Malawi Liverpool Wellcome Programme, Blantyre, Malawi; Institute of Infection, Veterinary and Ecological Sciences, University of Liverpool, Liverpool, United Kingdom; Institute of Infection, Veterinary and Ecological Sciences, University of Liverpool, Liverpool, United Kingdom; Institute of Infection, Veterinary and Ecological Sciences, University of Liverpool, Liverpool, United Kingdom; Institute of Infection, Veterinary and Ecological Sciences, University of Liverpool, Liverpool, United Kingdom; Department of Genetics, University of Cambridge, Cambridge, United Kingdom; World Health Organization, Geneva, Switzerland; Malawi Liverpool Wellcome Programme, Blantyre, Malawi; Institute of Infection, Veterinary and Ecological Sciences, University of Liverpool, Liverpool, United Kingdom; Department of Medical Laboratory Sciences, School of Life Sciences and Health Professions, Kamuzu University of Health Sciences, Blantyre, Malawi; Ministry of Health, Government of Malawi, Lilongwe, Malawi; Institute of Infection, Veterinary and Ecological Sciences, University of Liverpool, Liverpool, United Kingdom; Malawi Liverpool Wellcome Programme, Blantyre, Malawi; Institute of Infection, Veterinary and Ecological Sciences, University of Liverpool, Liverpool, United Kingdom; Department of Medical Laboratory Sciences, School of Life Sciences and Health Professions, Kamuzu University of Health Sciences, Blantyre, Malawi; Malawi Liverpool Wellcome Programme, Blantyre, Malawi; Institute of Infection, Veterinary and Ecological Sciences, University of Liverpool, Liverpool, United Kingdom

**Keywords:** diarrhea, health care-seeking behavior, Malawi, *Shigella*, surveillance

## Abstract

**Background:**

Malawi is among 7 countries participating in the Enterics for Global Health (EFGH) *Shigella* surveillance study, which aims to determine the incidence of medically attended diarrhea attributed to *Shigella*, a leading bacterial cause of diarrhea in children in low-resource settings.

**Methods:**

We describe the EFGH study site in the densely populated informal settlement of Ndirande Township, Blantyre, Malawi. We explore the site’s geographical location, demographic characteristics, and the healthcare-seeking behavior of its population, particularly for childhood diarrhea. We also describe the management of childhood diarrhea at the health facility, and the associated challenges to attaining optimum adherence to local and national guidelines at the site.

**Conclusions:**

Our overarching aim is to improve global health through understanding and mitigating the impact of diarrhea attributed to *Shigella*.

## Teaser

Malawi is participating in the Enterics for Global Health (EFGH) study to determine the incidence of diarrhea attributed to *Shigella* in children aged <5 years. This article describes the study site in Ndirande Township, Blantyre, Malawi.

Diarrhea due to the gram-negative bacteria *Shigella* is responsible for approximately 60 000 deaths annually among children <5 years old in low- and middle-income countries. In addition to causing acute bloody and nonbloody diarrhea, *Shigella* is associated with persistent and prolonged diarrhea, elevated inflammatory markers linked to environmental enteric dysfunction, and linear growth faltering [1, 2].

In Malawi, childhood diarrhea is recognized as a significant health concern with incidence likely to increase due to climate change. The Global Burden of Diseases study has estimated that over 6.51% of all deaths in Malawi were due to diarrheal disease in 2019 [3]. The EFGH study aims to determine the incidence of *Shigella*-attributable medically attended diarrhea to guide decision making for clinical trials of *Shigella* vaccines. This article provides an overview of the Ndirande study site in Blantyre, Malawi.

## MALAWI COUNTRY PROFILE AND HEALTH SYSTEM

**Figure 1.**
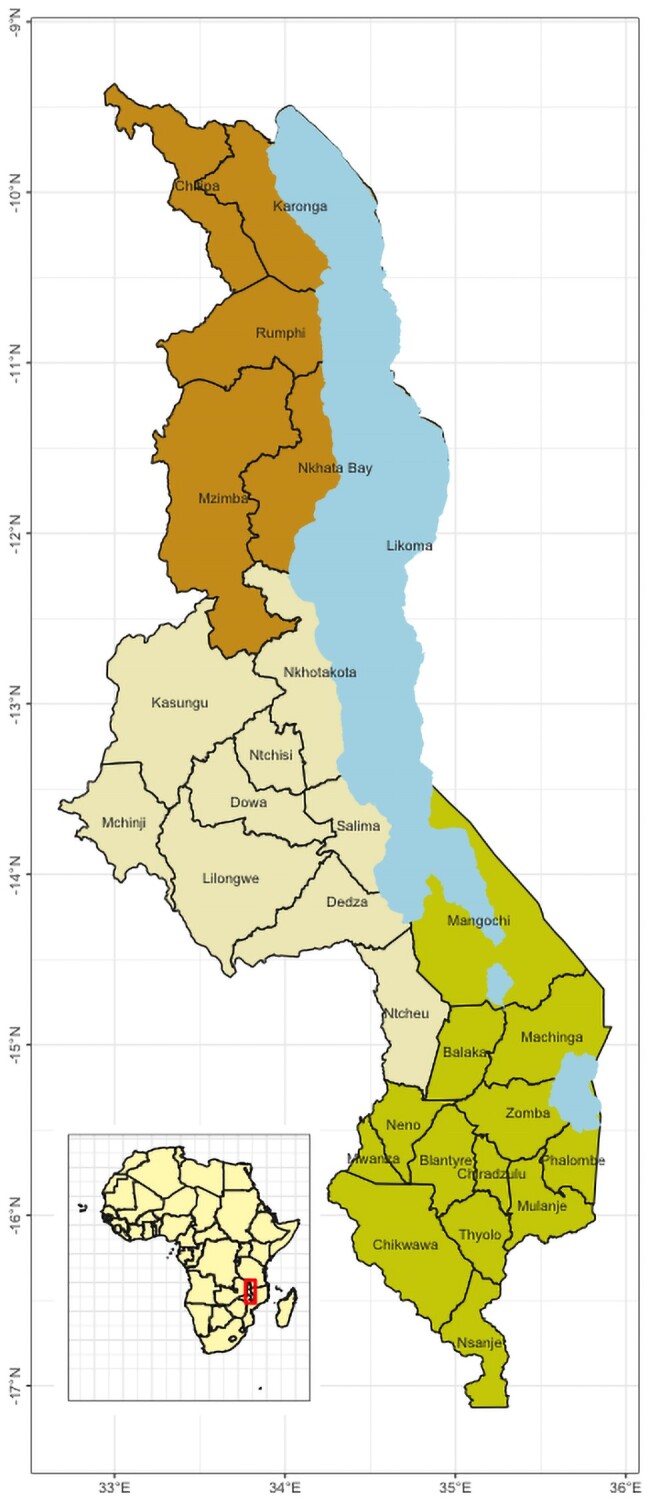
Map of Malawi.

Malawi is a landlocked country in the southeastern region of Africa, bordered by Tanzania, Mozambique, and Zambia (Figure 1). It has a total area of 118 484 km^2^, 20% of which is covered by water (mostly Lake Malawi). The lowest and highest points of the land lie at 37 and 3003 meters above sea level, respectively. Consequently, the climate pattern in Malawi is highly variable, with temperatures averaging from 14°C to 32°C [4]. Malawi has 3 seasons: hot-wet, hot-dry, and cool-dry. From May to August, the weather is cool and dry, becomes hot in September and October, and the hot-wet (rainy) season begins in October or November, continuing until April. The wet season is usually associated with increased infectious diseases, including diarrhea. The country is divided into 3 regions, namely the Northern, Central, and Southern regions. There are 28 districts in the country: 6 districts in the Northern region, 9 in the Central region, and 13 in the Southern region (Figure 1). The country had an estimated population of 17.6 million people in the 2018 census, with an average annual growth rate of 2.9%, with an estimated population of 20.4 million people by 2022 [5]. According to 2019 World Bank estimates, the gross domestic product per capita is approximately 645.84 US dollars [6].

Malawi operates a 3-tiered health system with primary, secondary, and tertiary levels interlinked through a referral system. The primary tier provides general medical care through community and rural hospitals and maternity units. The secondary level comprises district hospitals and acts as a referral level for the primary level; it provides specialized care, including laboratory diagnostics and rehabilitation services. The tertiary level provides healthcare services for conditions that require highly specialized care. The Ministry of Health (MOH) is Malawi's primary overseer of healthcare and healthcare providers. The government provides 63% of health services, the Christian Health Association of Malawi (CHAM) provides 26%, and the remainder is provided by private for-profit providers [7]. The curative and preventive healthcare services in the MOH facilities are free, while CHAM and private facilities have user fees.

## CHILD HEALTH AND IMMUNIZATION SCHEDULE

Malawi has taken significant strides toward protecting child health. According to United Nations Children’s Fund estimates, its under-5 mortality rate is 42 per 1000 live births, and the infant mortality rate is 31 per 1000 live births as of 2021 [8].

**Figure 2.**
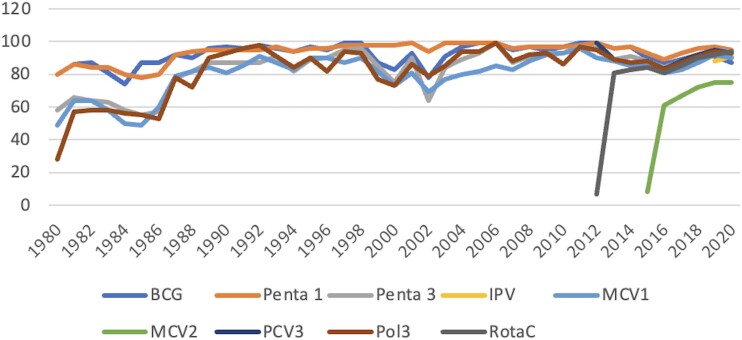
Malawi coverage estimates of most vaccines (1980–2020). Abbreviations: IPV, inactivated polio vaccine; MCV, measles containing vaccine; PCV, pneumococcal conjugate vaccine; Penta, pentavalent; Pol, polio vaccine 3; RotaC, rota virus conjugate vaccine.

Malawi's commitment to child health is exemplified by its robust immunization program. The Expanded Programme on Immunization (EPI), established in 1979, reached the universal immunization goal within a decade, with 80% coverage for all vaccine antigens. This high immunization coverage has been sustained over the years (Figure 2).

Malawi has recently introduced several new vaccines to its EPI; these include diphtheria, pertussis, tetanus, hepatitis B, and *Haemophilus influenzae* type b vaccines (2002), 13-valent pneumococcal conjugate vaccine (2011), rotavirus vaccine (2012), a second dose of measles vaccine (2015), measles-rubella vaccine (2017), inactivated polio vaccine (2018), human papillomavirus vaccine (2019), and most recently, the typhoid conjugate vaccine (2023). The country is also piloting the RTS,S malaria vaccine. The current immunization schedule is shown in Table 1. Lessons from past experiences indicate that several vital factors have facilitated the successful integration of new vaccines into Malawi's health system; efficient strategic planning, collaboration, and coordination between policymakers, health professionals, and international partners are instrumental in overcoming potential barriers to vaccine introduction. Additionally, high levels of vaccine acceptance also serve a key role in successful vaccine introduction [9].

**Table 1.** Malawi Immunization Schedule

Age	Vaccine
At birth or first contact	BCG
At birth up to 2 wk	OPV0
At 6 wk	OPV1, DPT-HepB-Hib1, PCV1, and Rota1
At 10 wk	OPV2, DPT-HepB-Hib2, PCV2, and Rota2
At 14 wk	OPV3, DPT-HepB-Hib3, PCV3, IPV
At 5, 6, and 7 mo	Malaria vaccines
At 6 mo and every 6 mo up to 59 mo	Vitamin A (children)
At 9 mo	Measles-rubella 1, TCV
At 15 mo	Measles-rubella 2
At 22 mo	Malaria vaccines
At 9–14 y	HPV vaccine
At first contact (15–45 y and pregnant women)	TT1
At 4 wk after TT1	TT2
At 6 mo after TT2	TT3
At 1 y after TT3	TT4
At 1 y after TT4	TT5
Within 2 wk of delivery	Vitamin A (postnatal mothers)

Abbreviations: DPT, diphtheria-pertussis-tetanus; HepB, hepatitis B; Hib, *Haemophilus influenzae* type b; HPV, human papillomavirus; IPV, inactivated polio vaccine; OPV, oral polio vaccine; PCV, pneumococcal conjugate vaccine; Rota, rotavirus; TCV, typhoid conjugate vaccine; TT, tetenus toxoid vaccine.

## INSTITUTION PARTNERSHIP OVERVIEW AND HISTORY

The Malawi-Liverpool-Wellcome Programme (MLW) was established in 1995. Located in Blantyre, MLW is strategically positioned next to Queen Elizabeth Central Hospital, the largest hospital in Malawi, and near the nation's largest medical school. The MLW is formed of a partnership of 3 organizations: the Kamuzu University of Health Sciences (formerly known as the University of Malawi College of Medicine), the Liverpool School of Tropical Medicine, and the University of Liverpool.

MLW initially focused on malaria research, but its portfolio has expanded over the last 2 decades, embodying the mission of conducting internationally excellent research for health benefits and nurturing the next generation of researchers. It hosts 15 research groups spanning 6 themes: infection biology, population health, maternal health, vaccines, clinical and experimental medicine, and social sciences.

Research at MLW is underpinned by the robust infrastructure provided by its 5 research support units: clinical research, data, statistical, laboratory, and the policy unit. The laboratory support unit provides high-quality, Good Clinical Laboratory Practice and International Organization for Standardization 15189–compliant laboratory support to all research projects at MLW, in both molecular and microbiology laboratories. The policy unit supports policy translation aspects of the projects conducted at the institution. Alongside, operations departments including finance and facilities further streamline the institution's research efforts.

## SITE DESCRIPTION

### Ndirande Township

**Figure 3.**
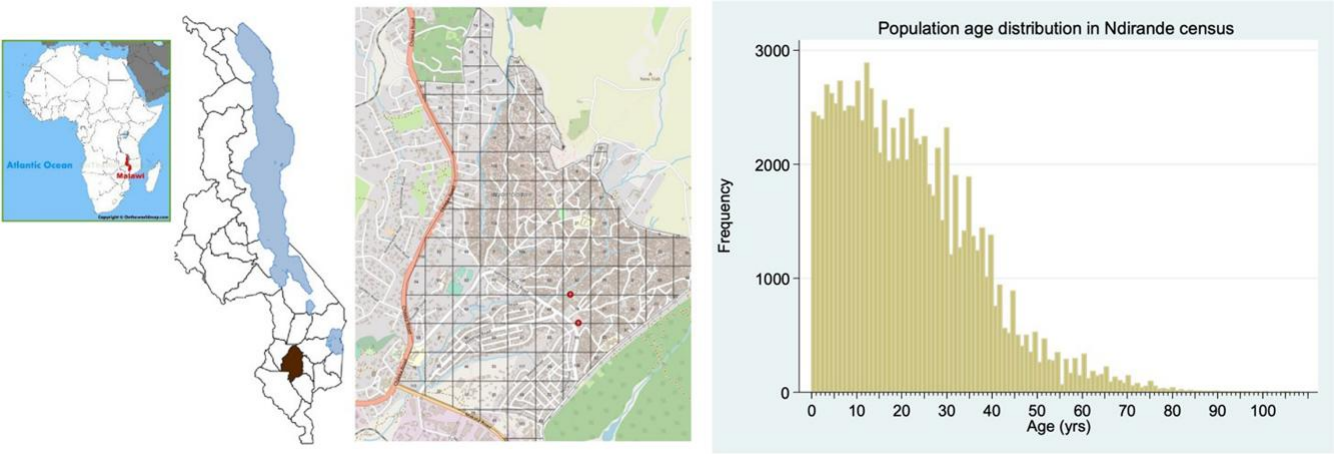
Map showing Ndirande Health Centre catchment area and age distribution.

The EFGH *Shigella* surveillance study will be conducted in Ndirande Township, one of several densely populated urban and periurban settlements in Blantyre District, southern Malawi (Figure 3). Ndirande exhibits the health, social, and economic challenges commonly found in Malawi's low-income residential areas, including undernutrition and limited access to clean water and sanitation facilities [10]. Ndirande is Malawi's oldest urban informal settlement, covering approximately 3.3 km^2^. It is the largest low-income residential area in the country [10]. The age-stratified population of Ndirande was enumerated by the Strategic Typhoid Alliance Across Africa and Asia (STRATAA) consortium in 2016 and updated in 2019 [11]. This latest household census recorded 23 765 households, estimating the total population size of Ndirande to be 103 497 (95% confidence interval [CI], 102 881–104 114). The population age distribution of Ndirande includes 0–4 years: 13 455 (95% CI, 13 375–13 535); 5–14 years: 27 427 (95% CI, 27 263–27 590); ≥15 years: 62 616 (95% CI, 62 243–62 989); and 2–24 months: 4761 (95% CI, 4733–4789) (Figure 3).

### Ndirande Health Centre

The Ndirande Health Centre (NHC), located in the heart of Ndirande, is instrumental in providing healthcare services to the local community and is the recruitment site for the EFGH study. Ndirande is 5 km from MLW.

The NHC provides various outpatient clinical services to the local community, addressing the most common childhood illnesses, including respiratory infections, diarrheal diseases, malaria, and other febrile infections. Notably, the NHC also provides emergency obstetric services, such as immediate surgical interventions, as a vital lifeline for pregnant women and newborns in the district.

The NHC's proximity to the community makes it a convenient access point for potential study participants relative to a tertiary care medical facility. This, in combination with NHC's comprehensive healthcare services, provides a conducive environment for participant engagement and retention in research studies.

### Management of Diarrheal Disease at Ndirande Health Centre

The data obtained from government records at NHC reveal that among the cases of diarrhea in children aged <5 years recorded at NHC from 1 January 2017 to 31 December 2019, 28% were identified as bloody diarrhea. When adjustments were made for healthcare utilization rates, the findings indicated that in children <5 years, the incidence rate of nonbloody diarrhea was 659.2 episodes (95% CI, 655.3–663.2) per 1000 person-years observed. In contrast, the incidence rate of bloody diarrhea was determined to be 259.9 episodes (95% CI, 258.4–261.5) per 1000 person-years observed. The EFGH study team works closely with healthcare providers and the MOH staff at NHC, fostering collaborative clinical care for participants and adherence to standard management guidelines (Tables 2 and 3).

**Table 2.** Site-Specific Guideline for Management Dehydration

Classification	Site-Specific Guideline
Dehydration classification	
Severe	Plan C: Ringer’s lactate solution (or Normal/saline) 30 mL/kg in 60 min (age <12 mo)/30 min (age ≥12 mo), then 70 mL/kg in 5 h (age <12 mo)/2.5 h (age ≥12 mo). Reassess every 15–30 min. Give ORS (5 mL/kg/h) as soon as the child can drink.
Some	Plan B: ORS dose age/weight table. Reclassify dehydration after 4 h and continue with A, B, C plans.
None	Plan A: Increase food and fluid intake to prevent dehydration.
Malnutrition classification	
Severe	Plan C: Give ReSoMal 5 mL/kg every 30 min for the first 2 h. Then, if the child is still dehydrated, give ReSoMal 5–10 mL/kg/h. Alternate with F-75, up to a maximum of 10 h.
Some	Plan B: Assume that all children with watery diarrhea have dehydration and prescribe ReSoMal as indicated above.
All children	Zinc supplementation for 10–14 days: 10 mg per day (age ≤6 months) or 20 mg per day (age >6 mo).

Abbreviations: ORS, oral rehydration solution; ReSoMal, rehydration solution for malnutrition.

**Table 3.** Site-Specific Guideline for Management of Dysentery or *Shigella*

Population	Site-Specific Guideline
Dysentery or *Shigella* upon culture confirmation	Azithromycin 15 mg/kg (max 1000 mg) on day 1, then 10 mg/kg (max 500 mg) for 4 days (first line). Ciprofloxacin 15 mg/kg per dose (max 500 mg) twice daily (caution in age <18 y) (second line). If no improvement at 48 hours: IV ceftriaxone 50 mg/kg once daily for 2 to 5 days q2–5d. If still no improvement consider amebiasis: metronidazole 10 mg/kg (max 750 mg) three times a day for 5 days
Suspected cholera (age ≥2 y + severe dehydration + cholera present in area)	Azithromycin 20 mg/kg (max 1000 mg) stat, or erythromycin 12.5 mg/kg four times a day for 3 days. Treat guardian with doxycycline 300 mg oral once dose stat.

Abbreviations: IV, intravenous; PO, oral.

## CHALLENGES WITH DIARRHEA MANAGEMENT GUIDELINES

While efforts to manage diarrhea at NHC have been significant, specific challenges persist in delivering guideline-adherent care. One of the critical challenges lies in supply chain management. Often, NHC experiences stock-outs of essential drugs and supplies, such as antibiotics, oral rehydration solution, and zinc supplements. These intermittent stock-outs undermine the effective implementation of diarrhea management guidelines and may result in suboptimal treatment options. To address this, the EFGH study provides backup World Health Organization–indicated treatments when there are stock-outs at the facility.

A shortage of trained healthcare personnel exacerbates suboptimal adherence to guidelines. The extremely low healthcare worker to-population ratio (reported to be 3.4 per 10 000 for nurses and 0.2 per 10 000 clinicians or physicians by the World Bank [6] for the year 2019 against the required threshold of 22.8 per 10 000) can result in overburdened healthcare facilities, potentially compromising the quality of care.

## HISTORICAL *SHIGELLA* INCIDENCE, PREVALENCE, AND ANTIMICROBIAL RESISTANCE DATA

*Shigella* species pose a significant public health threat in Malawi, particularly impacting children aged <5 years. Our diarrhea surveillance to date has focused primarily on children with watery, nonbloody diarrhea at the Queen Elizabeth Central Hospital and district health centers in Blantyre. These findings showed that *Shigella* was present in 15.8% of hospitalized cases (which included those with dysentery) and 5.7% of asymptomatic community controls [12]. Antimicrobial resistance testing was performed using a panel of 20 antibiotics; all *Shigella* isolates were resistant to trimethoprim-sulfamethoxazole and ampicillin but susceptible to the other antibiotics tested [13]. More recently, however, whole genome sequencing of *Shigella* isolates from invasive infection in Malawi has identified the emergence of fluoroquinolone resistance [14]

## HISTORICAL HEALTHCARE-SEEKING FOR DIARRHEA

Understanding healthcare-seeking behavior is important for contextualizing incidence estimates. Existing data from Malawi indicate that a high proportion of caregivers seek medical attention when a family member, particularly a child <5 years old, suffers from diarrhea. A study survey conducted in the Chikwawa district of Malawi in 2007 provides insights into the healthcare-seeking behaviors of the population; this survey showed that 67% of the participating women reported taking their children aged <5 years to a healthcare facility when they experienced a diarrhea episode [15].

Similarly, the 2015 Demographic and Health Survey from the guardians of >17 000 children across Malawi found that 66.8% (2393/3584) of children reportedly suffered from an episode of diarrhea in the 2 weeks preceding the survey. Strikingly, 66.8% of the 2393 cases had sought treatment or medical advice. This underscores the common healthcare-seeking behaviors of Malawian caregivers for childhood diarrhea [16].

These studies together reflect a tendency toward healthcare engagement in childhood diarrhea, underlining the role of health facilities as crucial points for intervention and data collection. However, it is worth noting that while two-thirds of caregivers sought medical advice during diarrheal episodes, one-third did not. This suggests there may be barriers to healthcare access, such as socioeconomic factors, logistical challenges, or knowledge gaps, which might hinder a complete utilization of healthcare services. Thus, in addition to disease prevention and treatment strategies, efforts should be made to understand further and address these barriers to maximize healthcare-seeking behaviors and ensure the health and well-being of all children in Malawi.

## TRAINING AND CAPACITY BUILDING

The EFGH Malawi site is deeply committed to supporting early career investigators and fostering equity, diversity, and inclusion. We provide mentorship, training, and research collaboration opportunities in enteric infections, allowing early career investigators to participate significantly in the study. Several of the investigators in the study are undertaking training at either Master’s or PhD level. Alongside this, we strive to cultivate an inclusive research environment that appreciates individual differences in gender, race, ethnicity, nationality, and socioeconomic background. Promoting fairness, equal opportunities, and representation of traditionally underrepresented groups in research activities and decision-making processes is a core part of our mission. Through these efforts, we aim to contribute to a supportive and balanced research community within and beyond the EFGH project.

## CONCLUSIONS

The EFGH study in Malawi aims to understand the burden of *Shigella* in an urban, low-resource setting. The Ndirande Township site in Blantyre, Malawi, with its known demographic characteristics and well-documented care-seeking behavior, provides an ideal setting for the surveillance study. The robust immunization program and the potential to incorporate a *Shigella* vaccine show promise for disease control. As the lead implementer, MLW is committed to diversity, equity, and inclusion, ensuring that we foster diverse voices and perspectives. This work, integrated with our overarching aim to improve global health, underscores the potential for significant contributions to understanding and mitigating the impact of *Shigella*-related diarrhea.

## Notes

***Acknowledgments.*** We acknowledge the Ministry of Health Malawi, including the Blantyre District Health Office, for supporting the conduct of the study at their facilities. We also thank parents who are allowing their children to be recruited in the study.

***Ethics statement.*** The study has been approved by the College of Medicine Research Ethics Committee that approves studies for the Kamuzu University of Health Sciences (reference number P./10/21/3437) and the University of Liverpool Ethics Committee (reference number 10596).

***Disclaimer.*** The views expressed are those of the author(s) and not necessarily those of the National Institute for Health and Care Research, the Department of Health and Social Care, the UK government, or the UK Health Security Agency.

***Financial support*.** This work was supported by the Bill & Melinda Gates Foundation (grant numbers INV-031791, INV-016650, INV-028721, INV-041730). N. A. C. is a National Institute for Health and Care Research (NIHR) Senior Investigator (NIHR203756) and is affiliated with the NIHR Global Health Research Group on Gastrointestinal Infections at the University of Liverpool; and the NIHR Health Protection Research Unit in Gastrointestinal Infections at the University of Liverpool, a partnership with the UK Health Security Agency in collaboration with the University of Warwick.

***Supplement sponsorship.*** This article appears as part of the supplement “Enterics for Global Health (EFGH) *Shigella* Surveillance Study-Rationale and Methods,” sponsored by the Bill & Melinda Gates Foundation.

## References

[1] Kotloff KL, Nataro JP, Blackwelder WC, et al Burden and aetiology of diarrhoeal disease in infants and young children in developing countries (the Global Enteric Multicenter Study, GEMS): a prospective, case-control study. Lancet 2013; 382:209–22. 23680352 10.1016/S0140-6736(13)60844-2

[2] Platts-Mills JA, Taniuchi M, Uddin MJ, et al Association between enteropathogens and malnutrition in children aged 6–23 mo in Bangladesh: a case-control study. Am J Clin Nutr 2017; 105:1132–8. 28381477 10.3945/ajcn.116.138800 PMC5402031

[3] Institute for Health Metrics and Evaluation . Global Burden of Disease (GBD). Available at: https://www.healthdata.org/research-analysis/gbd. Accessed 7 August 2023.

[4] Vincent K, Dougill AJ, Mkwambisi DD, Cull T, Stringer LC. Analysis of existing weather and climate information for Malawi. 2014. Available at: https://futureclimateafrica.org/wp-content/uploads/2015/02/Malawi-existing-Analysis-of-existing-weather-and-climate-information-for-Malawi-final-with-disclaimer.pdf. Accessed 7 August 2023.

[5] National Statistical Office . 2018 Malawi population and housing census. Preliminary report. Zomba, Malawi: National Statistics Office; 2018.

[6] The World Bank . GDP per capita (current US$)—Malawi. 2021. Available at: https://data.worldbank.org/indicator/NY.GDP.PCAP.CD?locations=MW. Accessed 6 July 2023.

[7] Government of the Republic of Malawi . Health sector strategic plan II 2017–2022. Lilongwe: Government of the Republic of Malawi; 2016.

[8] United Nations Children’s Fund . Malawi—demographics, health and infant mortality. Available at: https://data.unicef.org/country/mwi/. Accessed 6 July 2023.

[9] Malawi Ministry of Health . Malawi’s strategy to reach immunisation zero dose children. Unpublished 2022.

[10] National Statistical Office . 2018 Malawi population and housing census main report. 2019. Available at: http://www.nsomalawi.mw/images/stories/data_on_line/demography/census_2018/2018 Malawi Population and Housing Census Main Report.pdf. Accessed 27 July 2022.

[11] Darton TC, Meiring JE, Tonks S, et al The STRATAA study protocol: a programme to assess the burden of enteric fever in Bangladesh, Malawi and Nepal using prospective population census, passive surveillance, serological studies and healthcare utilisation surveys. BMJ Open 2017; 7:e016283. 10.1136/bmjopen-2017-016283 PMC5726077 28674145

[12] Iturriza-Gómara M, Jere KC, Hungerford D, et al Etiology of diarrhea among hospitalized children in Blantyre, Malawi, following rotavirus vaccine introduction: a case-control study. J Infect Dis 2019; 220:213–8. 30816414 10.1093/infdis/jiz084 PMC6581894

[13] Phiri AFND, Abia ALK, Amoako DG, et al Burden, antibiotic resistance, and clonality of *Shigella* spp implicated in community-acquired acute diarrhoea in Lilongwe Malawi. Trop Med Infect Dis 2021; 6:63. 33925030 10.3390/tropicalmed6020063 PMC8167763

[14] Stenhouse GE, Jere KC, Peno C, et al Whole genome sequence analysis of *Shigella* from Malawi identifies fluoroquinolone resistance. Microb Genom 2021; 7:000532. 33945457 10.1099/mgen.0.000532 PMC8209728

[15] Masangwi S, Ferguson N, Grimason A, Morse T, Kazembe L. Care-seeking for diarrhoea in southern Malawi: attitudes, practices and implications for diarrhoea control. Int J Environ Res Public Health 2016; 13:1140. 27854311 10.3390/ijerph13111140 PMC5129350

[16] National Statistical Office Malawi and ICF . Malawi Demographic and Health Survey 2015–16. 2017. Available at: https://dhsprogram.com/pubs/pdf/FR319/FR319.pdf. Accessed 27 July 2022.

